# THE IMPACT OF DEPRESSION ON MATERNAL RESPONSES TO INFANT FACES IN PREGNANCY

**DOI:** 10.1002/imhj.21538

**Published:** 2015-11-09

**Authors:** J.A. Macrae, R.M. Pearson, R. Lee, D. Chauhan, K. Bennert, A. Burns, H. Baxter, J. Evans

**Affiliations:** ^1^ Exeter University Bristol

**Keywords:** depression, maternal responsiveness, self report, pregnancy, prenatal, perinatal, actitud de respuesta materna, auto reporte, embarazo, prenatal, perinatal, dépression, réaction maternelle, auto rapport, grossesse, prénatal, périnatal, Depression, mütterliche Responsivität, Selbstbericht, Schwangerschaft, pränatal, perinatal, 抑うつ(状態), 母親の応答性, 自己報告, 妊娠, 出産前, 産期, 抑鬱症, 產婦的響應速度, 自我報告, 懷孕, 產前, 圍產期。

## Abstract

Research has suggested that prenatal depression may be associated with disrupted maternal responses to infant stimuli, with depressed pregnant women not showing the bias toward distressed infants as that observed in nondepressed pregnant women. The current study examined the effects of depression on self‐ reported responses to infant stimuli, in early pregnancy. Women with clinical depression (*n* = 38), and nondepressed women (*n* = 67) were recruited from a wider cognitive behavioral therapy trial. They completed Maternal Response Scales in which they were presented with images of distressed, neutral, and happy infant faces, with no time limit. The women rated their responses to these images along three dimensions—wanting to comfort, wanting to turn away, and feelings of anxiety—using Likert scales via a computerized task. There was evidence that women with depression in pregnancy showed different responses than did women without depression. Women with depression were substantially more likely to be in the highest quartile for ratings of wanting to turn away, odds (OR) ratio = 4.15, 95% confidence intervals (CIs) = 1.63–10.5, *p* = .003, and also were substantially less likely to be in the highest quartile for wanting to comfort a distressed infant face, OR = 0.22, 95% CIs = 0.09–0.54, *p* < .001. Findings are consistent with there being both a heightened avoidant and a reduced comforting response toward distressed infants in depressed pregnant women, providing some support that depression disrupts maternal preparations at a conscious level.

## RESUMEN

**Trasfondo**: La investigación sugiere que la depresión prenatal pudiera estar asociada con los trastornos en las respuestas maternas a las manifestaciones de estímulo del infante, sin que las mujeres embarazadas deprimidas muestren los prejuicios hacia los infantes angustiados que se observan en las mujeres embarazadas no deprimidas. El presente estudio examinó los efectos de la depresión en las respuestas auto‐reportadas a las manifestaciones de estímulo del infante al principio del embarazo.

**Método**: Se reclutaron mujeres con depresión clínica (n = 38) y mujeres no deprimidas (n = 67) de un grupo mayor de experimento de terapia de conducta cognitiva (CBT). Las mismas completaron las Escalas de Respuesta Materna (MRS) en las cuales se les presentaron imágenes de caras de infantes angustiados, neutrales y felices sin límite de tiempo. Las mujeres evaluaron sus respuestas a estas imágenes a lo largo de tres dimensiones; querer calmar, querer alejarse, y sentimientos de ansiedad, usando las escalas Likert 1–5 por medio de una tarea computarizada.

**Resultados**: Se dio evidencia de que las mujeres con depresión en el embarazo mostraron diferentes respuestas que aquéllas sin depresión. Las mujeres con depresión estuvieron sustancialmente más propensas a quedar en la más alta cuarta parte de puntajes de querer alejarse (promedio de posibilidades 4.15, (95% de intervalos de confianza (CI) = 1.63‐10.5), p = 0.003), y estuvieron también sustancialmente menos propensas a quedar en la más alta cuarta parte por querer calmar la cara de un infante angustiado (promedio de posibilidades 0.22 (95% de intervalos de confianza (CI) = 0.09‐0.54), p<0.001).

**Conclusiones**: Los resultados son consistentes con la presencia tanto de una elevada propensión a evitar y una reducida respuesta a calmar hacia los infantes angustiados en el caso de mujeres embarazadas deprimidas, lo cual provee algún apoyo a que la depresión altera las preparaciones maternas a un nivel consciente.

## RÉSUMÉ

**Contexte**: Les recherches suggèrent que la dépression prénatale peut être liée à des réponses maternelles aux stimuli du bébé interrompues, avec des femmes enceintes déprimées ne faisant pas preuve de parti pris envers des bébés en détresse observés chez des femmes enceintes non déprimées. Cette étude a examiné les effets de la dépression sur les réactions au stimuli du bébé auto‐rapportées, au début de la grossesse.

**Méthode**: Des femmes atteintes d'une dépression clinique (n = 38), et des femmes non déprimées (n = 67) ont été recrutées au moyen d'une étude plus grande sur la thérapie cognitivo‐comportementale (TCC). Elles ont rempli les Echelles de Réaction Maternelle (abrégé en anglais MRS) dans lesquelles on leur a présenté des images de visages de bébés en détresse, neutres, et heureux, sans limite de temps. Les femmes ont évalué leurs réactions à ces images au moyen de trois dimension: voulant réconforter, voulant se détourner, et sentiments d'anxiété en utilisant les échelles Likert 1–5 au moyen d'un exercice informatique.

**Résultats**: Il n'y a eu aucune preuve que les femmes atteintes de dépression durant la grossesse ont fait preuve de réactions différentes que les femmes sans dépression. Les femmes atteintes de dépression étaient bien plus à même de se trouver dans le quartile le plus élevé de notations “voulant se détourner” (rapport de cote 4,15, (95% d'intervalles de confiance (IC) = 1,63‐10,5), p = 0,003) et étaient bien moins à même de se trouver dans le quartile le plus élevé de notations “voulant réconforter un bébé en détresse” (rapport de cote 0,22, (95% (d'intervalles de confiance IC) = 0,09‐0,54), p<0,001).

**Conclusions**: Ces résultats correspondent au fait qu'il existe à la fois une réaction d’évitement intensifiée et une réaction réduite de réconfort envers les bébés en détresse chez les femmes déprimées, soutenant que la dépression perturbe les préparations maternelles à un niveau conscient.

## ZUSAMMENFASSUNG

**Hintergrund**: Untersuchungen zeigen, dass pränatale Depression mit einer gestörten mütterlichen Reaktion auf Impulse des Säuglings in Verbindung gebracht werden kann, indem depressive schwangere Frauen im Vergleich zu nicht‐depressiven schwangeren Frauen bei beunruhigten Säuglingen nicht mit Befangenheit reagieren. Die aktuelle Studie untersucht die Auswirkungen der Depression auf selbstberichtete Reaktionen auf Impulse des Säuglings in der frühen Schwangerschaft.

**Methode**: Frauen mit klinischer Depression (n = 38), und nicht‐depressive Frauen (n = 67) wurden aus einer größeren CBT‐Studie rekrutiert. Sie füllten die Maternal Response Scales (MRS) aus, wobei ihnen Bilder von beunruhigten, neutralen und glücklichen Gesichtern von Säuglingen ohne Zeitlimit präsentiert worden. Die Frauen bewerteten diese Bilder im Rahmen einer computergesteuerten Aufgabe mithilfe einer 1–5 Likert‐Skala bezüglich drei Dimensionen: Wunsch zu beruhigen/trösten; Wunsch sich abzuwenden und Gefühle von Angst. Ergebnisse Es gab Evidenz dafür, dass Frauen mit Depression in der Schwangerschaft andere Reaktionen als Frauen ohne Depression zeigen. Frauen mit Depression waren mit einer wesentlich höheren Wahrscheinlichkeit im höchsten Quartil für die Bewertung sich abwenden zu wollen (Odds Ratio 4.15 (95% Konfidenzintervall (CI) = 1.63 bis 10.5), p = 0.003) und mit einer wesentlich geringeren Wahrscheinlichkeit im höchsten Quartil für den Wunsch, einen beunruhigten Säugling zu trösten (Odds Ratio 0.22 (95% Konfidenzintervall (CI) = 0.09‐0.54), p <0.001). Schlussfolgerungen Die Ergebnisse stimmen mit der Annahme überein, dass bei depressiven schwangeren Frauen sowohl eine erhöhte vermeidende und eine reduzierte tröstliche Antwort auf beunruhigte Säuglinge zu beobachten ist. Dieses Ergebnis wiederum liefert Unterstützung dafür, dass Depression die mütterlichen Vorbereitungen auf einer bewussten Ebene stört.

## 抄録

背景　出産前の抑うつは、乳児の刺激への母親の応答の中断を伴うかもしれないこと、そして抑うつ的ではない妊婦に観察された苦痛の乳児へのバイアスを、抑うつ的な妊婦は示さなかったことが、研究から示唆されている。方法　臨床的に抑うつ状態の女性(n = 38)と、抑うつ的ではない女性(n = 67)が、広範なCBT調査から集められた。彼女たちは、母親応答尺度Maternal Response Scales, (MRS)を終わらせた。その検査では、彼女たちは、時間制限なしに苦痛、中性、および幸せな乳児の表情のイメージを提示された。女性は、それらのイメージに対する自分たちの応答を、コンピュータ化した課題で、3次元、すなわち慰めたい、逃げ出したい、および不安を感じる、に沿って、1‐5のリッカート尺度を用いて評価した。結果　妊娠中に抑うつ状態が明らかだった女性は、抑うつのない女性に比べて、異なる応答を示した。抑うつ状態の女性は、「逃げ出したい」の評価が最高位4分の1にいることが、実質的により多く(オッズ比 4.15, (95% 信頼区間 (CI) = 1.63‐10.5), p = 0.003)、また、乳児の苦痛の表情に対して「慰めたい」の最高位4分の1にいることが実質的により少なかった(オッズ比 0.22, (95% 信頼区間(CI) = 0.09‐0.54), p<0.001)。結論　これらの所見は、抑うつ状態の妊婦において、高い回避と苦痛な乳児への慰め反応の低下があるという所見と一致していて、抑うつ状態が意識レベルで母親の準備を妨げている事を支持する。

## 摘要

背景。研究顯示, 產前抑鬱症可能與母親對嬰兒刺激物的破壞反應有關, 抑鬱的孕婦沒有表現出非抑鬱孕婦對有苦楚嬰兒的偏差。本研究審查在懷孕初期抑鬱症對嬰兒刺激物反應自我報告的影響。 方法。從更廣泛的CBT試驗招募女性臨床抑鬱症患者(N = 38)和非抑鬱女性(N = 67)。她們完成了Materanl Response Scales(MRS), 在沒有時間限制下, 觀看苦楚, 中性和快樂的嬰兒圖片。婦女用電腦1‐5李克特量表,從三個方面評估她們對圖片的反應;想要安慰, 想要轉身離去, 和焦慮感覺。 結果。證據表明, 女性抑鬱症患者在懷孕期間比沒有抑鬱症的女性表現出不同的反應。女性抑鬱症病患者較高可能在評估想要轉身離開時, 得最高四分位數(勝算比4.15, (95%置信區間(CI) = 1.63‐10.5), p = 0.003)和較低可能在評估想安慰一個痛苦的嬰兒的臉, 得最高四分位數(比值比為0.22(95%信賴區間(CI) = 0.09‐0.54), p<0.001)。 結論。研究結果顯示, 抑鬱孕婦對有苦楚嬰兒有高度迴避和降低安慰的反應, 研究結果對抑鬱症破壞母親在意識層面的準備, 提供了一定的支持。

## Abstract (ar)

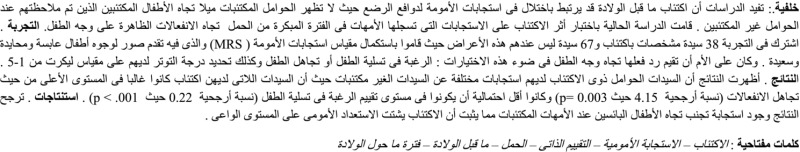

The effects of maternal depression on child outcomes are well‐documented, including its impact on child language development, cognitive functioning, and ongoing risk of psychosocial and emotional difficulties (Goodman et al., 2011). Although it is unknown exactly how depression has an impact on child outcomes possible mechanisms include: shared heritability, mediation through stressful life events or mediation through aspects of parenting such as exposure to negative maternal cognitions, affect and behaviours (Goodman et al., 2011; Goodman & Gotlib, 1999). A growing body of research has begun to examine the influence of maternal responsiveness on mother–child interactions, the importance of how attentive to infant‐related stimuli the mother is, and how motivated she is to respond. Subsequently, a number of studies have found that postnatal depression is associated with disrupted mother–infant interactions (Murray, Fiori‐Cowley, Hooper, & Cooper, 1996) through low maternal responsiveness (defined as a low attraction or motivation toward anything infant‐related (Pearson, 2010).

Although research to date has focused on maternal responsiveness postnatally, there is increasing evidence from both animal and human studies to suggest that maternal responsiveness develops before birth and across pregnancy (Fleming, Steiner, & Corter, 1997; Maestripieri & Zehr, 1998). Studies also have indicated that sensitivity toward emotional faces and attentional biases toward infant stimuli develop across pregnancy (Pearson, Cooper, Penton‐Voak, Lightman, & Evans, 2010; Pearson, Lightman, & Evans, 2009, 2010). Importantly, the maternal responsiveness and mother–child attachment observed in pregnancy appears to be predictive of maternal sensitivity postnatally, indicating that the patterns observed in pregnancy may be indicators of later maternal responses (Pearson et al., 2011; Siddiqui & Hagglof, 2000; Kim, Park, & Shin, 2006; Thun‐Hohenstein, Wienerroither, Schreuer, Seim, & Wienerroither, 2008).

A number of research studies have linked maternal perinatal depression to insecure or disorganized mother–infant attachments (Campbell et al., 2004; Wan & Green, 2009). Although research looking at the developmental trajectories of such children is still at an early stage, it may be that attachment relationships mediate the transmission of “disorders” such as depression (Wan & Green, 2009). Evidence has suggested that maternal sensitivity or responsiveness is associated with attachment status (Ainsworth, 1979; Bigelow et al., 2010; Egeland & Farber, 1984; Isabella & Belsky, 1991; Meins, Fernyhough, Fradley, & Tuckey, 2001). It appears, then, that maternal responsiveness to infant needs is linked to, or predictive of, attachment security (Mills‐Koonce, Gariepy, Sutton, & Cox, 2008).

Maternal responsiveness is typically measured by either recording observed behavior or by measuring cognitive processing of related stimuli that are salient, such as infant faces. Evidence from observational studies have found differences in how the mother cradles her infant (Reissland, Hopkins, Helms, & Williams, 2009) as well as the amount of maternal eye gaze, maternal language, the amount of gesturing, and the level of coordinated attention between mother and child Campbell et al., 2007). Research also has indicated that women who have antenatal depression, as compared to nondepressed women, are less sensitive when playing with their baby (Murray et al., 1996).

In research using cognitive‐processing measures, infants’ faces are often used because they are considered to be “salient” stimuli for mothers. These are used to measure maternal sensitivity, as infant facial expression is an important nonverbal signal from the infant to the mother. Evidence also has suggested that the processing of infant faces is distinct from that of adult faces because infants have a different facial configuration (Lorenz, 1971; Stein et al., 2010), meaning that the measure is specific rather than just capturing changes in responses to faces in general.

When applying this approach to measuring maternal responsiveness during pregnancy, Pearson, Cooper et al., 2010 found that depressed pregnant women did not show an attentional bias toward distressed as compared with nondistressed infant faces whereas nondepressed pregnant women did show this bias. The results suggested that women with depression had a bias in the opposite direction (i.e., away from distressed infants), although the confidence intervals included no effect. It is not clear whether there is a true difference that would been seen only in a larger sample, and if so, whether this difference is due to an increased speed of the depressed women to disengage from distressed faces or due to a slower speed to disengage from nondistressed faces. This study focused on early cognitive processing, and previous studies of early cognitive processing have produced inconsistent results. The length of time that a stimulus is presented is thought to influence its emotional valence, with increased presentation time being associated with increased emotionality (Stein et al., 2010). For example, in a design utilizing later cognitive processing, Stein et al. (2010) found that when infant facial stimuli were presented for 200 ms as opposed to 100 ms, mothers rated the infant emotions as more extreme. Women with postnatal depression also rated “negative” infant faces more “negatively” than did nondepressed women. Although this study has indicated that mothers with depression may rate negative infant stimuli more negatively, the mother's own emotional responses to the infant facial stimuli are not clear. Processing of infant stimuli in the context of perinatal depression is complex, and it is possible that although these mothers may judge stimuli as negative, their own emotional responses may not be affected, such as wanting to comfort a distressed infant.

The current study aimed to examine self‐reported emotional responses to infant stimuli from early to midpregnancy (6–18 weeks at recruitment) in women with and without depression. Looking at women's self‐reported responses to stimuli early in pregnancy may help to develop an understanding of mothers’ interpretation of, and behavior toward, infant emotion. The study aimed to investigate whether the responses of depressed women were most consistent with a lack of reward (not wanting to comfort) or with an avoidant response (wanting to turn away). To control for the fact that a woman may not want to comfort because they are experiencing anxiety (avoidant response), measures of anxiety also were included to assess whether comforting responses were independent of anxiety responses. We hypothesized that pregnant women with depression would report heightened avoidance (turning away) and a reduced comforting response to the presentation of infant faces. Following previous evidence, we also hypothesized that this effect would be the strongest for distressed infant faces.

## METHOD

### Participants

A total of 176 pregnant women were referred to the study by community midwives in the North Bristol region at their booking appointments. They were initially recruited for a wider study aiming to validate a depression screening instrument, which also included a pilot cognitive behavioral therapy (CBT) trial. Women were invited to participate in the current study if they were able to fluently read English and were less than 18 weeks pregnant so that they did not exceed the eligibility window of 19 weeks by the time they had completed the study. They were excluded if midwives were aware of any current psychotic illness. A total of 379 responses were received from the midwives, including 71 (19%) who declined to take part in the study at booking. Of those agreeing to take part, 88 (29%) screened positive, and 220 (71%) negative for depression. Eighty women were excluded, and 52 declined after researcher contact (see Table 1), leaving 176 women. Only a subsample of the women who were originally recruited took part in the current study due to time limits. All participants gave informed consent. Of the 105 women who completed the Maternal Response Scales (MRS) designed for this study, 38 women were identified as depressed using the Clinical Interview Schedule‐Revised (CIS‐R; Lewis, Pelosi, Araya, & Dunn, 1992) according to International Statistical Classification of Diseases and Related Health Problems, 10th Edition (ICD‐10; World Health Organzation, 1992) diagnostic criteria (*n* = 38), and 67 women were identified as having no diagnosis of depression (*n* = 67). In the final sample, mean age of participants was 31 years, and mean weeks of gestation was 13. Primiparous women represented 47% of the sample. At the time of assessment, only 8 women were currently taking antidepressant medication, and 8 had stopped medication due to pregnancy. Full sample demographics are included in Table 1.

**Table 1:** Sample Demographics. Occupation Defined by the National Statistics Socioeconomic Classification System

Characteristic	Full Sample Recruited	Current Study Whole Sample	Current Study Depressive Symptom Group	Current Study Nonsymptom Group
	(*n* = 176)	(*n* = 105)	(*n* = 38)	(*n* = 67)
*M* age in years (range)	30 (18–44)	31 (19–44)	30 (20–41)	31(19–44)
*M* gestation in weeks (range)	13 (9–19)	13 (9–19)	14 (9–19)	13 (10–18)
Primiparous, *n* (%)		49 (47)	21 (55)	28 (41)
Occupation *n* (%)				
Higher Managerial/Professional	22 (13)	12 (11)	3 (8)	9 (13)
Lower Managerial/Professional	34 (19)	27 (26)	6 (16)	21 (31)
Intermediate	63 (36)	35 (33)	16 (41)	18 (27)
Small Employers	2 (1)	2 (2)	0 (0)	2 (3)
LowerSupervisory/Technical	7 (4)	3 (3)	1 (3)	2 (3)
Semi Routine	9 (5)	5 (5)	2 (5)	3 (4)
Routine	3 (2)	1 (1)	1 (3)	0 (0)
Unemployed	13 (7)	4 (4)	4 (11)	0 (0)
No Data for Occupation	23 (13)	16 (15)	5 (13)	12 (18)
Ethnicity, *n* (%)				
White Caucasian	161 (91)	105 (100)	38 (100)	67 (100)
Other	15 (9)	0 (0)	0 (0)	0 (0)

### Procedure

Between May 2010 and February 2011, women who were referred to the study by midwives during routine practice were visited at home by a researcher. The women were then asked to complete the CIS‐R (Lewis et al., 1992), the Edinburgh Postnatal Depression Scale (EPDS; Cox, Holden, & Sagovsky, 1987), a questionnaire regarding demographic and pregnancy information, an attention bias task not reported here (for a full description and previous findings, see Pearson, Cooper et al., 2010), and the MRS. The women always were asked to complete the attention bias task before the MRS task, in case the conscious processing involved in the MRS task influenced women's responses in the attention bias task, which involves early cognitive processing. Ethical approval was given by the Southmead National Health Service Research Ethics Committee and North Bristol Trust, as part of a wider CBT trial (09/H0102/75).

### Measures

#### The CIS‐R (Lewis et al., 1992)

The CIS‐R is widely used with community samples and is a computerized, self‐administered interview that generates severity scores on a scale of 0 () to 4 () for 14 classified neurotic symptoms encompassing both anxiety and depression (Lewis et al., 1992). These symptoms then cluster according to ICD‐10 criteria. The interview is fully standardized and is as reliable as the Composite International Diagnostic Interview (CIDI; World Health Organization, 1992) (Robins, Wing, & Wittchen et al., 1998). It also is as reliable when conducted by a lay or trained interviewer (Lewis et al., 1992). The CIS‐R uses a total symptom score of 12 or above to classify individuals as having a clinically significant mental health disorder. Because some of the CIS‐R symptoms classifying a diagnosis of depression also can be problematic indicators in pregnancy because they involve physical symptoms, the EPDS also was completed, which does not include these.

#### The EPDS

The EPDS (Cox et al., 1987) is a self‐report questionnaire that screens for postnatal depression symptoms by avoiding the use of physical prognostic indicators. The EPDS is rapid to use and has high reliability and validity for both postnatal and antenatal women (Cox et al., 1996). The EPDS uses a symptom score from 9 to 12 to classify mild to moderate depressive symptoms and over 13 to classify major depressive symptoms (Cox et al., 1987).

#### The MRS

A measure of self‐reported emotional response to infant facial expressions was designed and developed for this study. Ten pregnant or recently pregnant women were consulted as to relevant emotional language to use on the measure, and the scales were piloted for understanding of language and ease of use. Previous use of similar scales in the research literature (Stein et al., 2010) was examined, and the scales were amended after piloting to be on a scale from 1 (low) to 8 (high) to be a more sensitive measure that is able to detect subtle variations in emotional self‐report.

**Figure 1:**
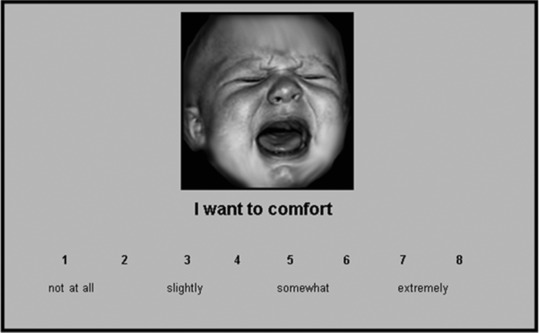
MRS Wanting to Comfort Scale with a distressed infant face (not to size). Scales were developed along three axes. Wanting to comfort (reward), wanting to turn away (avoidance), and feelings of anxiety.

Administering the MRS involved presenting distressed, neutral, and happy infant face images (seven of each). These images were randomized and presented in e‐Prime Professional 2.0 software (Psychology Software Tools, Pittsburgh, PA), with no time limit, to allow for the stimuli to be consciously processed. Conscious processing in this kind of task is thought to take effect after 500 ms (R.P. Cooper, 2010). Mean time taken in this study was 197,536 ms (for full reaction times, see Table 2). Under each picture, one of three MRS Likert scales was presented (see Figure 1): I want to comfort, I want to turn away, and I feel anxious. Each image was presented three times, in random order, each time with one of the scales presented underneath the pictures (anxiety, comforting, turning away). Participants were asked to press the number key that felt the most relevant to them for each picture and scale. To reduce social desirability effects, participants were encouraged to press the most meaningful response for them and were told that no answer was right or wrong.

**Table 2:** Reaction Times (seconds) to Response Scales Between Depressed and Nondepressed Conditions

Condition	Depressed			Nondepressed		
*n* =	37			68		
Response Scale	Want To Comfort	Want To Turn Away	Anxiety	Want To Comfort	Want To Turn Away	Anxiety
*M*	2.04	2.09	2.04	1.1	2	1.94
*SD*	0.79	0.82	0.83	1.01	1.1	1.10
Minimum–Maximum Time	1.01–4.6	1.05 –4.58	0.94 – 4.41	0.92–8.1	0.88–8.1	0.82–8.26

**Figure 2:**
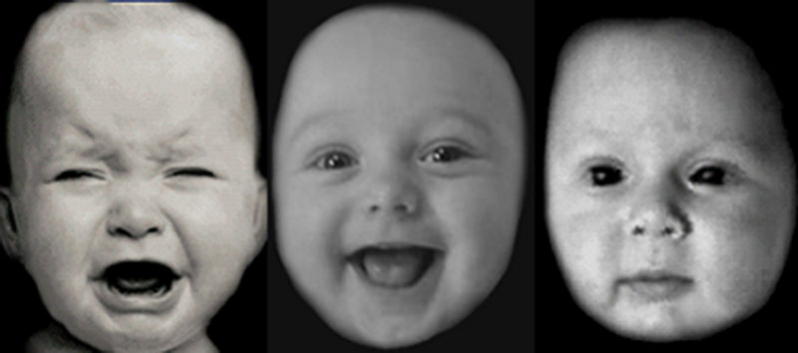
Distressed, neutral, and happy infant faces (Taken from Pearson et al., 2010).

The infant faces used in this study were those developed by Pearson, Cooper et al. (2010). The pictures were cropped, converted into gray scale, and matched to adult faces (Ekman & Friesen, 1971) for brightness positioning and contrast. Pictures included distressed, neutral, and happy faces (Figure 2).These images were piloted on nonpregnant women to test for emotional valence, and participants were forced to make a decision regarding whether the infant was distressed, neutral, or happy. Mean accuracy for this was 90 to 100%. These images have been validated in previous studies examining perinatal attentional processing (Pearson, Cooper et al., 2010), and were used with full permission from the authors.

### Analyses

The analyses for this study followed three stages. Following from our hypotheses, Stage 1 examined the relationship between depression and the primary responses of turning away and wanting to comfort, for distressed infant faces. Stage 2 examined these responses toward neutral and happy faces. Stage 3 investigated whether there was an independent effect of stimuli showing infant distress on the responses of women with depression as compared to women who were not depressed.

For all analyses, the independent variable was depression (“depressed/nondepressed” according to CIS‐R classification), with the dependent variable being maternal response (The MRS). These analyses were conducted using ordinal logistic regressions, as the dependent variable (MRS responses) was converted into an ordinal variable because it was not normally distributed and could not be normalized using transformations. As such, the MRS data were grouped into quantiles to derive an ordinal variable. The data fit most evenly into quartiles, with close‐to‐equal frequencies. The quartiles and their score ranges are shown in Table 3. Single predictor unadjusted ordinal logistic models were initially run on the data. The models were then adjusted for parity and age.

**Table 3:** Score Ranges and %Depressed for Each Quartile

Response	Wanting To Comfort				Turning Away			
Quartile	1	2	3	4	1	2	3	4
Frequency	29	23	29	24	33	19	27	25
Depressed Frequency (%)	18	4	10	6	10	15	17	15
	(62.07)	(17.32)	(34.48)	(25)	(30.03)	(78.95)	(62.96)	(60)
Nondepressed Frequency (%)	11	19	19	18	23	14	20	10
	(37.93)	(82.61)	(65.51)	(75)	(69.7)	(73.68)	(74.07)	(40)
Minimum to Maximum Rating	1.8–4.3	4.3–6.1	6.1–7.1	7.2–8	1.0–1.0	1.1–1.6	1.6–2.7	2.8–7.6

All analyses were repeated using the EPDS. As results were comparable, we primarily present results using the CIS‐R, which is the more in‐depth and diagnostic instrument.

Ordinal logistic regression models examine the odds ratio (OR) of an individual scoring at one level of an ordinal variable compared to the lowest level. Generalized rather than simple ordinal logistic regressions were conducted, as the proportionality of odds assumption was violated. The generalized ordinal logistic regression models calculated the OR for being in each quartile of the MRS (compared to the lowest quartile) according to whether women were depressed.

Primary analyses investigated the relationship between depression and responses of wanting to comfort and wanting to turn away. Our primary analyses focused on these responses to distressed infant faces because previous evidence has suggested that depression appears to particularly affect responses to distressed infant stimuli (Pearson, Cooper et al., 2010). Turning away and anxiety responses were correlated (see correlation matrix, Table 4); however, we also investigated anxiety responses in secondary analyses.

**Table 4:** Correlation Matrix of Maternal Response Ratings

		Anxiety			Comforting			Turning away		
		Distressed	Happy	Neutral	Distressed	Happy	Neutral	Distressed	Happy	Neutral
Anxiety	Distressed	1.00								
	Happy	.4689**	1.0000							
	Neutral	.7637**	.7423**	1.0000						
Comforting	Distressed	−0.1290	−.2901*	−.0854	1.0000					
	Happy	−.2650*	.0474	−.1205	.3388**	1.0000				
	Neutral	−.1755	−.0705	−.0402	.5957**	.8146**	1.0000			
Turning Away	Distressed	.6199**	.5153**	.4232**	−.4187**	−.0640	−.2220	1.0000		
	Happy	.4410**	.8635**	.6357**	−.3764**	−.0162	−.1186	.5813	1.0000	
	Neutral	.4959**	.7064**	.5924**	−.3547**	−.0288	−.1475	.6806**	.8729**	1.000

*p < .05. **p < .001.

**Table 5:** Descriptive Statistics Across Conditions According to CIS‐R Classification

	Depressed			Nondepressed		
Faces	*M*	*SD*	Range	*M*	*SD*	Range
	Comforting					
Distressed	5.08	1.99	1.68–8.00	6.06	1.51	2.71–8.00
Neutral	3.74	1.79	1.00–8.00	4.12	1.83	1.00–8.00
Happy	3.19	1.83	1.13–8.00	3.17	2.12	1.00–8.00
	Turning away					
Distressed	2.76	1.85	1.85–7.57	1.80	1.08	1.00–7.14
Neutral	1.89	0.95	1.00– 5.17	1.39	0.60	1.00–4.00
Happy	1.60	0.94	1.00– 5.29	1.20	0.38	1.00–2.57
	Anxiety					
Distressed	3.68	1.84	1.00– 7.43	2.67	1.52	1.00–6.86
Neutral	2.45	1.33	1.00–6.57	1.70	0.79	1.00–4.14
Happy	1.84	1.16	1.00– 6.14	1.23	0.42	1.00–2.86

## RESULTS

### Impact of Depression on Turning Away to Distressed Faces

**Figure 3:**
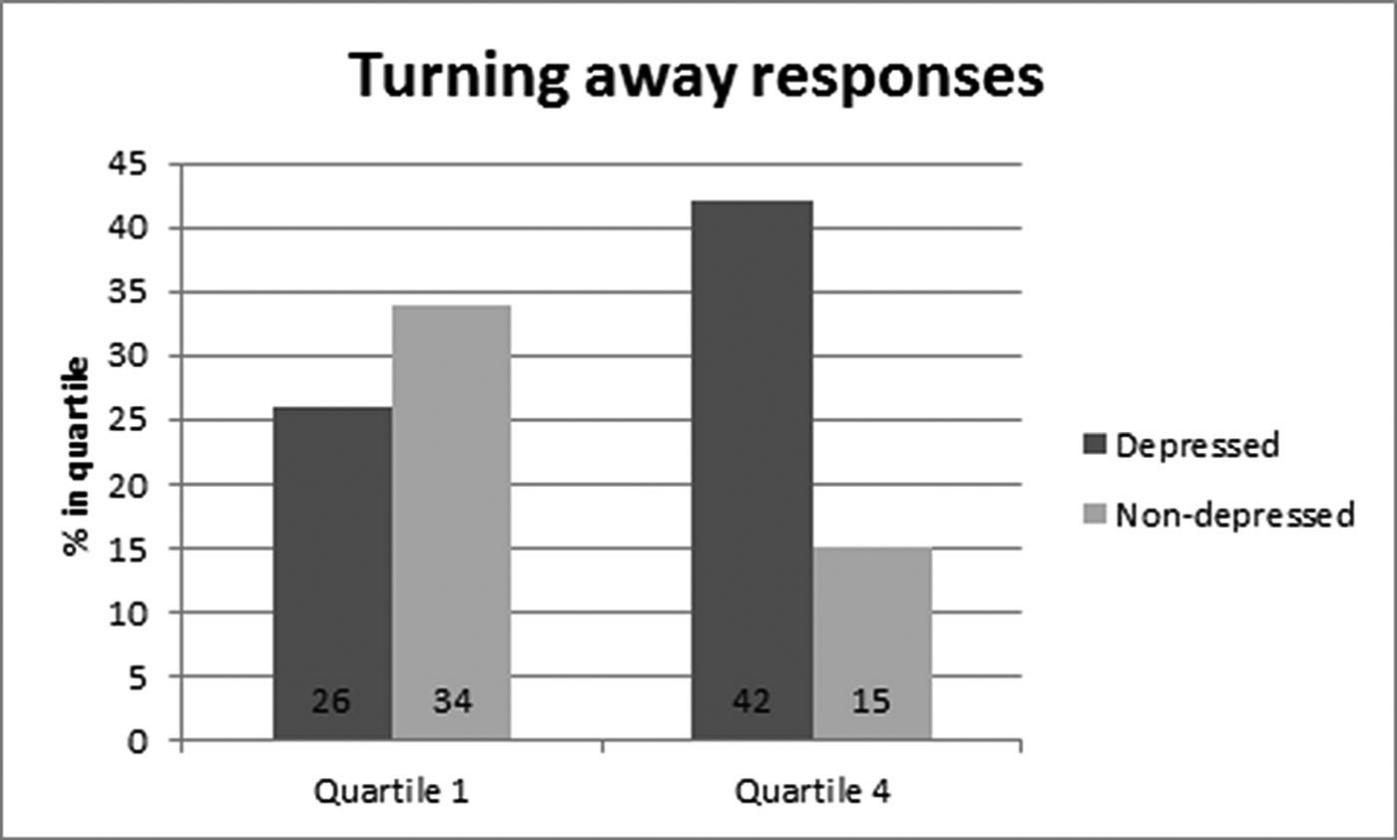
Turning away responses in depressed and nondepressed women to distressed infant stimuli. Confidence intervals Quartile 1—Depressed: 15–49%, Nondepressed: 51–84%. Confidence Intervals Quartile 4—Depressed: 41–80%, Nondepressed: 20–59.

The ordinal logistic regression model, adjusted for age and parity, provided evidence that depressed women were substantially *more likely* to be in the highest (vs. the lowest) quartile for reporting “wanting to turn away,” as compared to women without depression, OR = 4.15, *p* = .003, CI = 1.63–10.50. Depression did not influence the likelihood of being in the second or third quartile, as compared to the lowest quartile. The percentage of depressed and nondepressed women in Quartiles 1 and 4 is shown in Figure 3. Response patterns were reversed in depressed and nondepressed groups of women (see Table 5).

### Impact of Depression on Wanting to Comfort Distressed Faces

**Figure 4:**
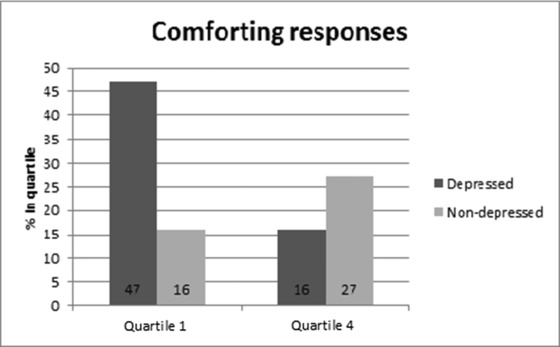
Wanting to comfort responses in depressed and nondepressed women toward distressed infant stimuli. Confidence intervals Quartile 1—Depressed: 42–72%, Nondepressed: 21–58%. Confidence Intervals Quartile 4—Depressed: 10–46%, Nondepressed: 53–90%.

The ordinal logistic regression model provided evidence that depressed women were significantly *less likely* to be in the highest (vs. lowest) category for wanting to comfort, OR = 0.22, *p* < .001, CI = 0.09–0.54. Depression did not influence the likelihood of being in Quartile 2 or 3, as compared to the lowest quartile. The percentage of depressed and nondepressed women in the lowest and highest quartiles (Figure 4) again shows a reversed trend for depressed and nondepressed women.

### Neutral Faces

The same response pattern for depressed and nondepressed women was observed for turning away to neutral faces, with depressed women being significantly *more likely* to be in the highest versus the lowest quartile for turning away, OR = 4.46, *p* = .04, CI = 1.59–12.49. However, the lack of a comforting response toward stimuli was found to be specific to distressed faces for women with depression, as there was a nonsignificant effect of depression on wanting to comfort neutral faces, OR = 0.72, *p* = .5, CI = 0.28–1.87.

### Happy Faces

There was a significant effect of depression on wanting to turn away from happy infant stimuli, with depressed women being *more likely* to be in the highest quartile for turning away, OR = 4.14, *p* = .003, CI 1.63–10.51, as compared to nondepressed women. However, there was no effect of depression on the likelihood of wanting to comfort happy faces, OR = 0.61, *p* = .33, CI = 0.23–1.64.

### Independent Effect of Depression on Distress

These models were run again in a combined model including responses toward distressed, neutral, and happy faces. This allowed us to explore whether there were any independent effects of depression on responses to distress specifically, over and above general responses toward infant faces. This model indicated that the effect of depression on women's desire to comfort a distressed infant remained following adjustments for their responses toward happy and neutral faces, OR = 0.16, *p* = .003, CI = 0.05–0.53. However, there was not an independent effect of depression on women's desire to turn away from a distressed infant; the effect diminished following adjustment for responses to neutral and happy faces, OR = 2.61, *p* = .091, CI = 0.86–7.92.

## DISCUSSION

To our knowledge, this is the first study investigating self‐reported responses to infant stimuli in prenatally depressed women. The study indicates that prenatally depressed women, in early–midpregnancy (8–19 weeks) already report an altered response pattern to infant stimuli, as compared to women who are not depressed.

Early in pregnancy, depressed women reported that they were much *more likely* to want to turn away from an infant when shown a picture of an infant face displaying any emotion and much *less likely* to want to comfort the infant when shown distressed infant faces specifically. This effect was independent of parity and maternal age.

Investigating responses in pregnancy is important, as there is evidence to suggest that pregnancy may be important in the development of emotional and behavioral responses to infants in preparation for the maternal role, and that depression may disrupt this. For example, research has suggested that depression in pregnancy is predictive of maternal ratings of child affect at 6 months’ postpartum (Huot, 2004). Previous studies also have examined early attentional processing of infant stimuli, which is potentially relevant to maternal responsiveness (Pearson, Cooper et al., 2010). In women with perinatal depression, later conscious processing of infant faces were examined, and showed that women with perinatal depression rate infant faces more extremely than do women without depression (Stein et al., 2010). The study reported here develops this further by investigating women's reports of their own responses to infant faces. One of the strengths of this study is its sample size: 105 women early in their pregnancies. Women with depression were overrepresented in the study (*n* = 38), as compared to the normal pregnant population, due to midwives preferentially referring women with depression to the study. Data on medication were available for only 83 of the 105 women; however, an even number of depressed and nondepressed women were currently taking or had, because of pregnancy, stopped taking antidepressant medication, so this small number is unlikely to have had an influence on the results. However, the nature of the recruitment strategy also allowed a large amount of demographic information to be collected, allowing for potentially confounding variables such as age and parity to be adjusted for in statistical analysis.

### Potential Mechanisms: The Understimulation or the Overstimulation Hypothesis

Two main hypotheses could explain the difference in responses to infant stimuli found in women with prenatal depression. The first hypothesis is that maternal responses may be hypersensitive and avoidant in women with depression. This is supported by studies that have indicated that individuals with depression show hypersensitivity to, followed by avoidance of, punishment and negative stimuli (Elliott, Sahakian, Herrod, Robbins, & Paykel, 1997; Eshel & Roiser, 2010). There is evidence that depressed women give more attention to and are more easily able to recognize distressed faces as well as misinterpret neutral or happy faces as distressed (Gur et al., 1992). The second hypothesis is that women with depression may be experiencing an understimulation of maternal responses due to impaired reward responses (Swain, Lorberbaum, Kose, & Strathearn, 2007). For example, there is evidence that depressed woman show reduced reward processing. They show anhedonia (the lack of a positive bias) as well as the reduced ability to learn a delayed reward association (Pizzagalli et al., 2009). In this way, it is possible that depression in pregnancy disrupts the learning of positive reward association toward infant faces. Functional magnetic resonance imaging studies also have suggested that women with depression show less brain activation in neural areas involved in reward processing in response to their own infant's cry than do women without depression (Laurent & Ablow, 2011).

This study aimed to explore further whether women with prenatal depression were more likely to report reduced reward responses (not wanting to comfort) or heightened avoidant responses (wanting to turn away) when presented with infant faces. Findings indicate that women with prenatal depression have a reduced response of both types; that is, both reduced reward responses and heightened avoidant responses to infant distressed faces, as compared to comparison nondepressed women.

It could be argued, however, that depressed women will show an altered response to almost any stimuli, and not specifically to infant stimuli. Importantly, within this study, although women showed both an increased avoidant and a lowered reward response to the distressed infant pictures, the reduced comforting responses were observed only for distressed infant faces, and not for neutral and happy faces. The strongest observed effect was reduced comforting responses, only seen in depressed women for distressed infant faces, indicating that women with depression during pregnancy specifically have a reduced desire to comfort distressed infants and are not simply unresponsive to infant faces in general. This can be contrasted to the heightened turning‐away response, which was seen in depressed women for all faces (distressed, neutral, and happy), indicating that women with prenatal depression were more likely to report heightened wanting to turn away from all of the infant stimuli regardless of level of distress. The fact that the depressed women in this study showed the strongest response to distressed infant faces antenatally is potentially highly significant for behavioral responses toward infants postpartum. These results provide further support to the idea that some aspects of maternal responsiveness may develop antenatally. Pregnancy provides a potential opportunity to intervene to improve outcome for mothers and their offspring.

### Limitations

The scales used in this research were developed specifically for the study and are a relatively simple measure of emotional self‐report. Although they have face validity, they have not been tested for construct validity; therefore, it cannot be assumed that a self‐report of not wanting to comfort indicates a lack of reward. In addition, as no further variables after parity and age were included in the analysis, it remains possible that other contextual factors such as marital conflict could influence MRS scores independently of depression. Although anxiety was accounted for using the MRS, diagnosis of anxiety using the CIS‐R was not adjusted for, which also may have influenced MRS scores. This topic would be useful to further research.

Limitations to this study also include the fact that emotional self‐report is prone to bias. For example, although not wanting to comfort was the strongest effect in this study, it is possible that women with depression felt that it was acceptable for them to report that they do not want to comfort but not acceptable to say that they want to turn away. Further limitations include that the infant images used in this design were not the women's own children. It could be argued that the women in this study's responses to unknown infant faces cannot be generalized to their responses and behaviors that they would show later in response to their own infants.

### Conclusion

This study indicates that early in pregnancy, women with depression are more likely to report wanting to turn away from all infant faces, whatever the emotion displayed. They also are specifically less likely to report wanting to comfort if the infant face appears distressed. The fact that this response was present so early in pregnancy has implications for the ways in which depressed mothers may interact with their infants behaviorally before and after birth. As this study sampled only women in the first and second trimesters of pregnancy, it also would be interesting to study women in their third trimester to investigate the development of these conscious maternal preparations.
